# The Application of Model-Based Systems Engineering to Rural Healthcare System Disaster Planning: A Scoping Review

**DOI:** 10.1007/s13753-023-00492-z

**Published:** 2023-05-24

**Authors:** Thomas A. Berg, Kelsi N. Marino, Kristina W. Kintziger

**Affiliations:** 1 College of Nursing, University of Tennessee-Knoxville, Knoxville, TN 37966 USA; 2 College of Public Health, University of Nebraska Medical Center, Omaha, NE 68198 USA

**Keywords:** Computer simulation, Disaster preparedness, Model-based systems engineering, Rural healthcare, Systems thinking

## Abstract

Disasters and other emergency events have complex effects on human systems, particularly if the events are severe or prolonged. When these types of events happen in rural communities, the resources of the local public health, healthcare, and emergency response organizations can be quickly depleted or overwhelmed. Planning for emergencies can help to mitigate their impact. Model-based systems engineering (MBSE) methods, including computer simulations, can provide insight on how best to prepare for these events and to explore the effects of varying approaches and resource utilization. To best apply these methods for improving disaster management in rural settings, a synthesis of the current body of evidence in this field is needed. The objective of this scoping review was to provide a descriptive overview of the application of computer simulation based on MBSE approaches to disaster preparedness and response for rural healthcare systems. Six studies met inclusion criteria, and varied in terms of MBSE method used, healthcare setting, and disaster type and context considered. We identified a gap in the research regarding the application of MBSE approaches to support rural healthcare disaster preparedness planning efforts. Model-based systems engineering and systems thinking, therefore, represent novel methods for developing tools and computational simulations that could assist rural communities better prepare for disasters.

## Introduction

It is well recognized that effective planning can improve disaster event response through emergency preparedness (Hyde et al. 2006; Skryabina et al. 2020). Despite the importance of disaster preparedness planning in improving outcomes, many rural communities are unable to plan adequately, possibly due in part to a lack of tools to help them determine the most effective use of limited resources, including those related to healthcare systems. Model-based systems engineering (MBSE)—the formalized application of modeling to systems (Ramos et al. 2012)—could be considered one such tool. The use of systems theory and computer simulation to describe systems behavior has already been proven useful for the healthcare (Chaffee and McNeill 2007), public health (Homer and Hirsch 2006; Moore et al. 2011), and disaster management sectors (Kapucu et al. 2009; Coetzee et al. 2016). However, before MBSE methods can be effectively applied to improving disaster preparedness and management for healthcare systems in rural settings, a synthesis of the current body of evidence is needed. An initial literature review indicated a gap in the research about this topic and prompted the authors to conduct this scoping review.

Healthcare systems play a pivotal role in disaster planning and response. While successful emergency preparedness planning requires considerable effort and resources from rural and urban communities, rural communities and their associated healthcare systems often have limited resources that can be quickly diminished or overwhelmed by a disaster event (Manley et al. 2006). Indeed, many rural communities experience a myriad of challenges that can hinder preparedness planning: limited resources (for example, equipment and supplies, training, and infrastructure), inadequate healthcare access for specialty services and higher care levels, remoteness and lack of transportation, low population density, communication issues, and socioeconomic factors (Edwards et al. 2008).

When planning for and responding to disaster events, rural communities rely on assistance from healthcare facilities, emergency medical resources, first responders (for example, fire and law enforcement), and local or regional public health departments. Unfortunately, many rural areas lack financial resources and local public health department staff, which limits the scope of services they can provide. Similarly, an increasing number of rural hospitals has resource constraints, which can prevent them from effectively supporting community disaster planning. Moreover, economic and staff resource pressures have forced an increasing number of rural hospital closures, with many remaining rural hospitals facing a high risk of closure (American Hospital Association 2022; Chatterjee et al. 2022; Sablik 2022). Further complicating this situation, nearly 20% of the U.S. population use rural hospitals as the primary point of healthcare access (Mason 2017; Rogan and Lewis 2020).

Though some rural communities near urban areas may have increased access to assistance and be able to leverage a broader range of resources in the event of a disaster, there remains challenges to reliance on traditional external resources. External (for example, federal) funding and disaster response assistance agreements are often hard to access or may be delayed for rural areas after a disaster (Kapucu and Rivera 2020). Additionally, large scale disasters (for example, that occur over wide geographic areas) can create competition between communities for limited resources or can completely cut off remote areas from available assistance.

Besides notable differences in resources in rural versus urban settings, it is also necessary to highlight important inequities between rural and urban healthcare systems that are based in economic, structural, and even cultural differences in healthcare delivery (van Dis 2002). Rural healthcare providers are more reliant on Medicare and Medicaid payments (Schlidt and Wilkinson 2021; Rural Health Info 2023), due to the generally older populations residing in their service areas, compared to urban healthcare providers. Further, rural areas are more dependent on community health workers to provide healthcare services than in urban areas due to rural provider shortages, and there are few mechanisms available for reimbursement of community health worker services (Rural Health Information Hub 2022). Even minor changes in payment-related policies can have a greater impact on the ability of rural providers to provide services. This can be exacerbated by limited staffing and information technology resources, with rural providers being less likely to meet the operational or regulatory requirements for changes in reimbursement models. Additionally, rural healthcare systems are limited by their representation and lobbying power in Congress (Heady 2002). The current focus is on providing health insurance to those in need rather than on rural healthcare infrastructure (Schlidt and Wilkinson 2021). Taken together, these challenges contribute to how effectively rural healthcare providers can plan for disasters and call for the development of innovative disaster preparedness tools to aid in such planning.

The concept of systems modeling can be applied to healthcare disaster preparedness planning and response, particularly when the relevant elements are considered complex, adaptive systems (Simonović 2010; HoseinDoost et al. 2019). Complex adaptive systems are characterized by the interaction of individual elements or agents on a micro-level in a dynamic and nonlinear manner that affects the system’s behavior (Carmichael and Hadžikadić 2019). The science of such systems focuses on the relationships between these elements, how they self-organize and sustain themselves, and how system behaviors emerge from these relationships (Chaffee and McNeill 2007; Coetzee et al. 2016; Preiser et al. 2018).

Model-based systems engineering builds on the foundation of systems engineering, using computer simulations to help understand the system’s dynamics, including the interaction of the component elements. It can increase the efficiency of disaster preparedness planning by allowing the planners to run “what if” scenarios, test assumptions, and understand the optimum allocation of limited resources. Furthermore, the unique complexities and challenges of disaster planning for rural healthcare systems can be modeled to demonstrate the effect of factors, such as communication and resource availability.

We choose scoping review methodology for this inquiry, as this type of review generates an overview of the current body of literature on a given topic, provides a summary of relevant results, and identifies gaps for future research. The fact that a scoping review does not include a critical appraisal of the literature (Arksey and O’Malley 2005) is beneficial for this research objective, as we expected the identified MBSE approaches, disasters, rural settings, and disaster contexts to be heterogeneous. Furthermore, completing a scoping review allowed us to have an expansive focus, while maintaining a comprehensive and rigorous approach to the topic.

The objective of this scoping review was to provide a descriptive overview of the application of MBSE-based computer simulation approaches to disaster preparedness and response for rural healthcare systems. The overarching purpose of this work was to support the development of MBSE tools and approaches to improve the ability of rural healthcare systems to respond to disaster related challenges, including consideration of the roles and relationships of other related systems. Model-based systems engineering methods can provide important insights on how best to prepare and plan for disasters in resource limited areas, like rural healthcare settings, and the resulting simulations can serve as a tool to explore the effects of varying approaches or interventions and resource utilization.

## Methods

The protocol for this scoping review followed Preferred Reporting Items for Systematic Review and Meta-Analysis (PRISMA) guidelines for reporting systematic review protocols (Moher et al. 2015) and conducting a scoping review (Tricco et al. 2018). The protocol was registered on Open Science Framework.^1^ We used Arksey and O’Malley’s (2005) framework and Tricco et al.’s (2018) more recent scoping review guidelines, specifically the PRISMA extension for scoping reviews (PRISMA-ScR). Five of the six stages of the Arksey and O’Malley (2005) scoping review framework were included: identifying the research question(s), identifying relevant studies, selecting studies for inclusion, charting the data, and summarizing and reporting. Due to time and resource limitations, we did not include the optional sixth stage of stakeholder consultation. The following seven research questions were used for this review.

1. What types of MBSE approaches have been used in disaster management in rural settings?
2. What types of disasters have been addressed with these methods?
3. In which healthcare settings have these methods been used?
4. To which disaster management cycle aspects have these methods been applied?
5. What measures have been used to assess the effectiveness of these methods (that is, which primary and secondary outcomes have been assessed)?
6. What were the real-world effects of using these methods in rural disaster management policy and planning?
7. What have been the strengths and limitations of using these methods for disaster management in rural healthcare settings?

We consulted with academic librarians in both the health sciences and engineering disciplines to choose electronic databases, define limits, and refine keywords. To obtain the most comprehensive body of evidence, we did not apply search limits (for example, publication types, years of publication, rural settings, and disaster types). A full set of Web of Science search terms is provided in Table 1.

**Table 1:** Full text search from Web of Science

Domain	Search Terms
Disasters	ALL=(disaster OR catastrophe OR “humanitarian operation*” OR “emergency management” OR “disaster management” OR “disaster planning” OR pandemic OR epidemic OR outbreak OR “natural disaster” OR pollution OR “agricultur* disaster” OR famine OR “agriculture* pests” OR “agriculture* diseas*” OR “environment* disaster” OR “environment* emergency” OR wildfire OR “wild fire” OR “wild land fire” OR “industrial disaster” OR “man-made disaster” OR terroris* OR war OR “political unrest” OR “industrial emerg*” OR “man-made disaster” OR “man-made emergency” OR “mass violence”)
Rural	ALL=(rural OR non-urban OR frontier)
Healthcare	ALL=(healthcare OR “health care” OR “healthcare system” OR “health care system”)
MBSE	ALL=(“decision science” OR “decision support system*” OR “multi-method model*” OR “multimethod model*” OR “multi-method simulation” OR “multi method simulation” OR “hybrid model*” OR “hybrid simulation” OR “discrete event simulation” OR “discrete event model*” OR “agent based model*” OR “agent-based model*” OR “system* dynamic*” OR “system* model*” OR “system* theory” OR “system* thinking” OR “computer model*” OR “computer simulation” OR “digital twin” OR “multi-level analys*” OR “multilevel analys*” OR “system* analys*” OR simulation OR “computational model*” OR “computational simulation” OR “equation-based model*” OR “equation based model*” OR “neural net*” OR “network simulation” OR “Bayesian model*” OR “Bayesian network*” OR “Monte-Carlo” OR “Monte Carlo” OR “Markov chain*” OR “quantitative model*” OR “artificial intelligence” OR “machine learning” OR “predictive analytics” OR “predictive model*”)
Outcomes	ALL=(resilience OR preparedness OR “disaster preparedness” OR “disaster response” OR “emergency preparedness” OR “emergency response” OR effectiveness OR mortality OR morbidity OR injury OR “economic losses” OR damage OR destruction OR death OR “lives lost” OR “disability-adjusted life years” OR “disability adjusted life years” OR “community function*”)

*MBSE* model-based systems engineering

The following databases were searched: Web of Science, CINAHL, Scopus, ACM Digital Library, Engineering Village, Business Source Complete, and PubMed. We also checked the reference lists of included studies to determine if anything was missed in our database searches. The eligibility criteria were English-language publications (journal articles, conference proceedings, books/chapters, and gray literature) that were published at any time, in any type of rural healthcare setting, in the context of any type of disaster, and included 10 of the most commonly used MBSE methods (computer simulation, agent-based models, system dynamics models, discrete event models, multi-method simulation, computational models, network models/simulation, Bayesian models, artificial intelligence, and machine learning).

Two authors served as reviewers and screened publication titles, abstracts, and full text. All disagreements were resolved either during discussions between the two reviewers or with expert assistance from the third author. Duplicates were deleted in a multi-step process based on publication title, journal, author, and other available information. Inter-rater reliability was calculated for each step of the screening process.

After the final set of publications was selected, the two reviewers extracted the following information for data charting: publication information, geographic context (for example, city, state, or country), study objectives/research questions, study type, MBSE approach used, healthcare setting (for example, clinic or hospital), disaster context (for example, tornado, flood, or pandemic), disaster management cycle context (for example, preparedness, response, recovery, or mitigation), sources and types of data used, evaluation and validation methods, model parameters (for example, supply chain, human resources, or patient volume), outcomes assessed (for example, morbidity or mortality), impact (for example, direct uses or application to policy or planning), and study strengths and limitations. We used these extracted data to conduct a qualitative assessment of charted data to identify themes in the included literature that promoted understanding of the topic. Data management was conducted using a combination of Microsoft Excel, EndNote reference software, and NVivo.

## Results

**Fig. 1:**
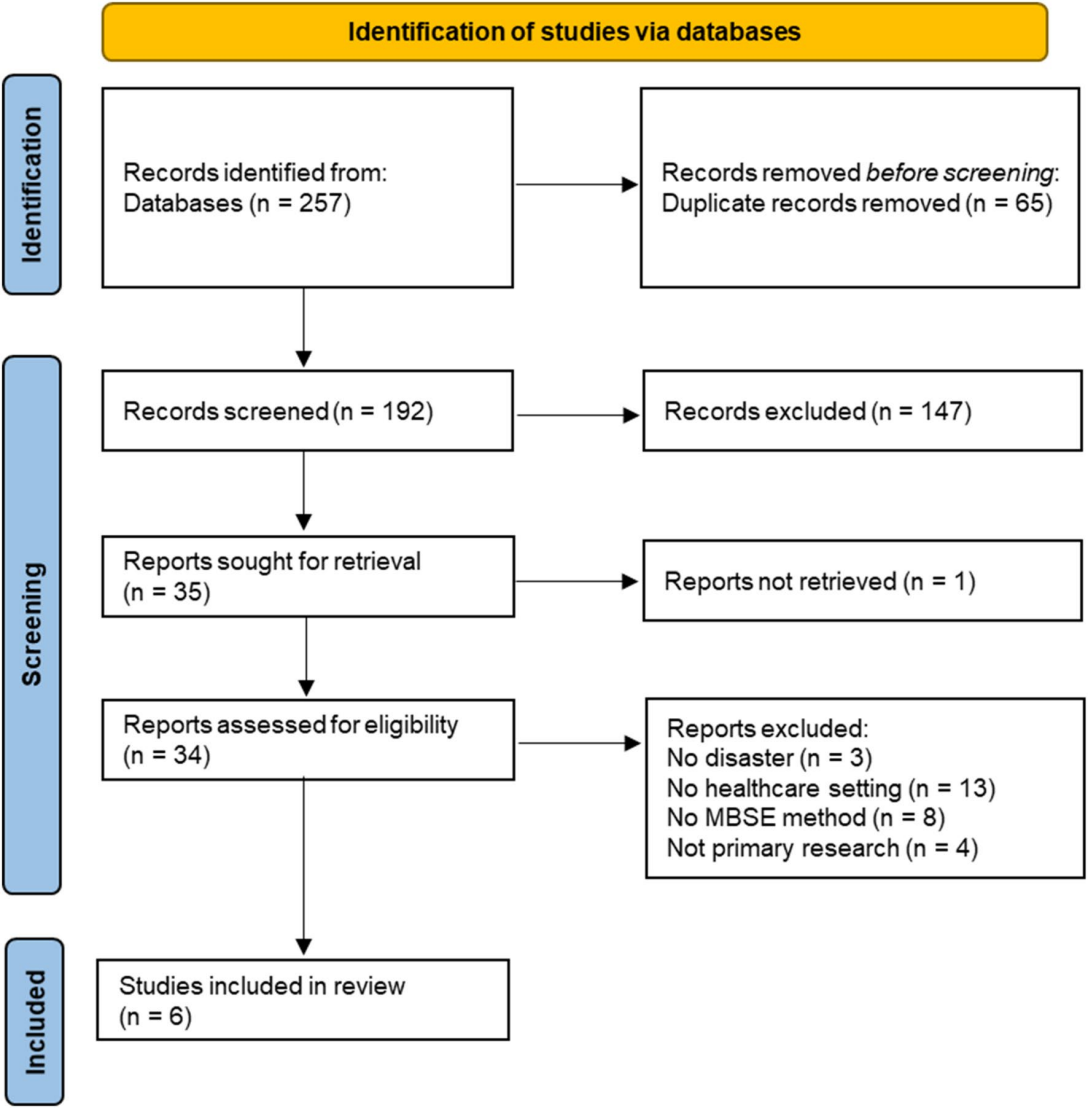
Literature search flow diagram

We identified 257 potential references from the database searches. After de-duplication, the two reviewers assessed titles and abstracts for 192 records, ultimately completing 34 full-text reviews. After review and discussion, we included only six primary studies in this scoping review. Inter-rater reliability was 83.9% for title/abstract screening and 88.2% for full-text screening. Details of the search flow with exclusion reasons are provided in Fig. 1. Characteristics of the included studies are shown in Table 2.

**Table 2:** Characteristics of included studies

Reference, Geographic Location	Study Objective(s)	MBSE Approach	Healthcare Setting	Disaster Context	Parameters or Inputs	Outcome(s)
Allen et al. (2020) Wessex, England	Support decision making for dialysis service delivery and dialysis patient transport in period prior to peak Covid-19 infection	Discrete event (dialysis provision) and Monte Carlo (vehicle routing) simulation	Dialysis centers throughout Wessex	• Biologic (Covid-19) • Response	• Proportion of infected patients • Time to infection • Time positive • Proportion requiring inpatient care • Time to admission • Length of stay • Mortality rate	• Change in outpatient and inpatient workload • Number of patients required to travel to different unit • Change in travel time
Chovatiya et al. (2019) Rajasthan, India	Predict epidemic dengue to support health system planning	Recurrent neural network	Indian Healthcare System	• Biologic (dengue) • Preparedness	• Weather (humidity, temperature, pressure, precipitation) • Air quality • Medical record for dengue patients	• Prediction of possible dengue outbreak
Kasturi et al. (2021) Indiana, United States	Determine the feasibility of using large statewide datasets to develop analytical models that can be used for population-health driven decision making related to Covid-19 healthcare resource utilization	Machine learning (gradient-boosted ensemble decision tree)	Indiana’s statewide Health Information Exchange	• Biologic (Covid-19) • Response	• Patient demographics • Diagnosis data • Past inpatient, outpatient, and emergency visit encounter history • Medications • Social determinants of health	• Hospitalization predictive ability at 1 week, 6 weeks, and across sub-populations
Patvivatsiri et al. (2007) Lubbock County and surrounding rural communities, Texas, United States	Investigate total time that patient stays in the healthcare system and hospital resource utilization during multiple hypothetical bioterrorism scenarios	Discrete event modeling	Trauma Service Area-B, which includes 10 hospitals/ clinics	• Bioterrorist events • Preparedness	• Arrival rates • Service times of treatment • Hospital resources (for example, doctors, nurses, beds, rooms) • Proportion of patient types (for example, cardiac, medical, pediatric)	• Average total time in system for each patient type • Utilization of resources in each treatment unit • Maximum surge capacity
Savitsky and Albright (2020) United States	Evaluate two different strategies for Covid-19 transmission prevention to labor and delivery healthcare workers	Decision analytic modeling with Monte Carlo simulation	Hospital labor and delivery units	• Biologic (Covid-19) • Response	• Probabilities of transmission given different parameters • Costs of testing and different types of personal protective equipment • Sensitivity and specificity of testing • Probabilities for different delivery scenarios	• Cost to prevent Covid-19 infection in one healthcare worker
Toerper et al. (2018) United States	Estimate hospital surge capacity before the occurrence of multiple-causality events	Monte Carlo simulation	Hospital	• All disaster types • Preparedness	• Space (for example, staffed/unstaffed beds convertible space) • Patients (for example, admissions of different types, transfers) • Disaster response strategies to activate (for example, bed expansion, reverse triage)	• Total number of disaster patients that can be accommodated • Admissions, discharges, and midnight patient census computed for each day

### Location

Four studies applied MBSE methods to disasters in the United States, including two that covered the entire United States (Toerper et al. 2018; Savitsky and Albright 2020) and one each focusing on a state (Kasturi et al. 2021) or a county and surrounding rural areas (Patvivatsiri et al. 2007). The two non-U.S. studies were conducted in Wessex, England (Allen et al. 2020) and Rajasthan, India (Chovatiya et al. 2019). None of the studies focused solely on rural areas, but rather rural areas were included along with urban areas as part of a larger geographic context.

### Model-Based Systems Engineering (MBSE) Methods

Monte Carlo methods were the most used approaches (Toerper et al. 2018; Allen et al. 2020; Savitsky and Albright 2020). Discrete event simulation/modeling (Patvivatsiri et al. 2007; Allen et al. 2020), recurrent neural network (Chovatiya et al. 2019), and decision-support science/modeling (Savitsky and Albright 2020; Kasturi et al. 2021) approaches were also used.

### Settings

We used a wide variety of healthcare settings as search terms. Hospitals were the most common setting, as they were included in some way in all six studies. Specific hospital units were mentioned in two studies: dialysis units (Allen et al. 2020) and labor and delivery wards (Savitsky and Albright 2020). Three studies included hospitals as part of larger service networks (Patvivatsiri et al. 2007; Chovatiya et al. 2019; Kasturi et al. 2021).

### Disaster Type and Management Cycle

The most frequent disaster type addressed was biological, including three Covid-19 studies (Allen et al. 2020; Savitsky and Albright 2020; Kasturi et al. 2021) and one dengue study (Chovatiya et al. 2019). One study examined bioterrorism events (Patvivatsiri et al. 2007) and one addressed all disaster types. The simulation studies were evenly split between preparedness and response applications.

### Simulation Model Inputs and Parameters

Parameters or inputs for simulation models varied greatly across studies and depended on the outcomes being assessed. Several studies focused on simulating disasters for hospital or health system resource planning. Allen et al. (2020) assessed the changes in outpatient and inpatient workloads and travel times in response to the Covid-19 pandemic. Kasturi et al. (2021) predicted hospitalization risk during Covid-19. Both Patvivatsiri et al. (2007) and Toerper et al. (2018) used simulations to predict maximum surge capacity during disasters, among other resource utilization outcomes. All four of these simulation studies used a variety of hospital utilization parameters and patient characteristics as model inputs.

To estimate the costs of preventing Covid-19 infections in healthcare workers, Savitsky and Albright (2020) used Covid-19 and labor and delivery parameters, as well as the costs associated with various testing and prevention strategies. Finally, Chovatiya et al. (2019) used patient characteristics and environmental data inputs to predict dengue outbreaks for healthcare system resource planning.

### Limitations

While the studies’ limitations varied (Table 3), they were frequently related to limited available data for model parameters or model assumptions that prioritized ease of use over flexibility. Particularly in the Covid-19 studies, simulations were limited both by the lack of available data early in the pandemic and the frequently changing epidemiology and knowledge of this emerging disease. Furthermore, the authors of two Covid-19-related studies acknowledged that their simulations did not address long-Covid or the long-term effects of Covid-19 infections (Savitsky and Albright 2020; Kasturi et al. 2021). Two studies did not present any model or simulation limitations (Patvivatsiri et al. 2007; Chovatiya et al. 2019).

**Table 3:** Transparency and validation/evaluation of included studies

Reference	Funding source	Conflicts of interest	Limitations	Limitations listed by authors	V&V	V&V method listed by authors
Allen et al. (2020)	Y	Y	Y	• Uncertainty about the spread of Covid • Certain model assumptions • Exclusion of home dialysis patients • Model choices prioritized speed and simplicity	Y	• Code review • Cross work • Expert review of model iterations • Modeling a range of scenarios
Chovatiya et al. (2019)	N	N	N	None reported	N	None reported
Kasturi et al. (2021)	N	Y	Y	• Limited data elements • Limited generalizability across different health systems • Excludes long Covid patients • Models trained using legacy patients • Long timeframe encompassing different pandemic waves and changing policies	Y	• Evaluated performance of each decision model with 20% holdout test dataset
Patvivatsiri et al. (2007)	Y	N	N	None reported	Y	Authors stated that the model was validated and verified but did not specify method used
Savitsky and Albright (2020)	Y	Y	Y	• Limited and changing data about current Covid pandemic • Did not consider visitors or support people • Did not consider long-term impacts of Covid	N	None reported
Toerper et al. (2018)	Y	N	Y	• Fixed assumptions used to ensure ease of use at the expense of flexibility • Limited data available on pediatric units • Does not include specialty units • Does not account for funds needed to resource the capacity or deal with damage caused to facility from event	N	None reported

*V&V* verification and validation, *Y* reported/stated, *N* not reported/not stated

### Model Evaluation

Half of the studies addressed model verification and validation (Patvivatsiri et al. 2007; Allen et al. 2020; Kasturi et al. 2021). However, only two studies specifically discussed simulation evaluation methods (Allen et al. 2020; Kasturi et al. 2021). Allen et al. (2020) used code review, cross model work, and expert opinion/review, while Kasturi et al. (2021) used training and holdout testing datasets (Table 3).

## Keywords

**Fig. 2:**
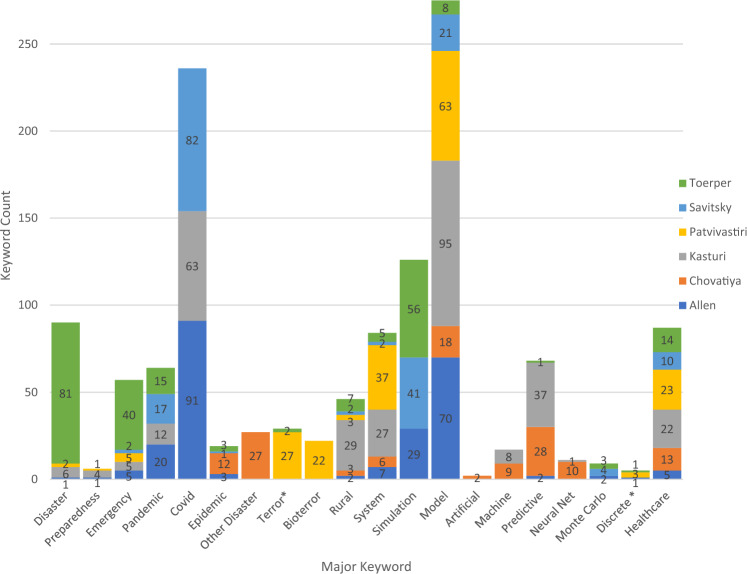
Keyword usage by publication

As part of this review, we analyzed the frequency of the primary keywords for each publication (Fig. 2). Considering the frequency of these keywords, there appear to be indirect references to using MBSE, but no explicit mention of its use. The use of certain MBSE techniques and methods was validated in the context of the research, specifically discrete event simulation, Monte Carlo modeling, neural net modeling, and predictive simulation.

The six articles identified included MBSE using different modeling approaches and included rural populations in a variety of contexts. These differences are highlighted as follows:

- Allen et al. (2020) used a combination of urban and rural areas using discrete event simulation of the Covid pandemic.
- Chovatiya et al. (2019) included urban, semi-urban, and rural populations for the simulation of disease propagation with artificial neural net systems modeling.
- Kasturi et al. (2021) contrasted rural and urban populations for consideration of general pandemic using a machine learning systems simulation.
- Discrete event simulation was also used by Patvivatsiri et al. (2007) focusing on bioterror effects on rural populations.
- Savitsky and Albright (2020) employed a decision analytic model for pandemics effects in rural areas.
- Monte Carlo systems modeling for general disaster effects was used by Toerper et al. (2018) for their rural population application.

## Discussion

Computer simulation is an ideal solution to advance disaster research because it considers disasters from a systems perspective, addresses uncertainties in the inputs by examining how the system behaves under different scenarios and provides decision-making support. Moreover, simulation can integrate qualitative and quantitative data and assimilate empirical data with existing interdisciplinary theories. It can also explain how interactions among agents or elements at multiple scales contribute to the emergent system behaviors across different levels (individual and community) or dimensions (preparedness and recovery; (Mostafavi and Ganapati 2019)).

For these reasons, Hoard et al. (2005) called for the use of systems modeling in rural disaster planning more than 15 years ago. However, despite the potential utility of this approach, minimal research has considered rural healthcare systems’ disaster preparedness as a complex adaptive system that considers the effects of changing social, political, economic systems and related environments and adaptive disaster responses. This point is illustrated by the fact that only six studies met this scoping review’s inclusion criteria.

This is not to say that many of the models that have been created and applied to urban, suburban, and other non-rural healthcare settings would not be relevant in the rural healthcare context. In fact, we identified several simulations and models that were developed and applied to urban healthcare settings that would be appropriate for use in rural settings, either in the existing form or with minor tweaks. Model-based systems engineering applications have become increasingly popular in large, urban healthcare systems, particularly related to pandemic response (Haghpanah et al. 2021; Possik et al. 2022). These applications have also found considerable use in other disaster contexts, including earthquakes (Arboleda et al. 2007; Jacques et al. 2014; Ceferino et al. 2020; Gul et al. 2020; Hassan and Mahmoud 2020), floods (Zehrouni et al. 2017), wildfire (Hassan and Mahmoud 2021), and other mass causality or surge-triggering events (Smith et al. 2009; TariVerdi et al. 2019; Benkacem et al. 2022; Trucco et al. 2022), and across the disaster management cycle, including preparedness (for example, evaluating healthcare systems planning (Smith et al. 2009; Zehrouni et al. 2017; Gul et al. 2020), assessing vulnerability (Arboleda et al. 2007)), response (for example, resource allocation, supply chain, and patient demand (Smith et al. 2009; Hassan and Mahmoud 2021; Benkacem et al. 2022; Trucco et al. 2022), or evaluate response activities or services (Jacques et al. 2014; TariVerdi et al. 2019; Ceferino et al. 2020)), or recovery (for example, recovery of healthcare systems (Hassan and Mahmoud 2020)). However, the focus of this scoping review is on the application of MBSE methods in rural healthcare settings, rather than the applicability of models to rural healthcare settings. Therefore, we did not include literature regarding the general applicability of models for rural healthcare in our review.

The overall search strategy for this review can be condensed into three parent keywords: rural, disaster, and MBSE. Because there are many definitions of “rural,” we chose not to focus on a particular definition, but instead considered the context in which the term was used. Similarly, there are many forms of disasters, most of which we attempted to include in our search. Our review focused on the contextual uses of the terms rural, disaster, and MBSE for each publication. These findings suggest that systems thinking—particularly MBSE—has not been widely used to support planning for rural healthcare disaster preparedness. Furthermore, while MBSE tools were used in the selected studies, it is not clear whether the strategy was employed with the explicit intent of applying a systems engineering approach or whether systems thinking was considered in the selection of a modeling method.

The results of this scoping review demonstrate that MBSE methods can be applied to improving disaster preparedness and response in rural healthcare settings. While MBSE methods are being more broadly used in disaster preparedness and response research, we identified few applications of MBSE methods in rural healthcare settings (Soyler and Sala-Diakanda 2010; Yaylali et al. 2014; Jackman and Beruvides 2019). Further, the applications of MBSE that we identified that included rural settings mostly included hospitals, either alone or as part of a system. While we did not identify notable differences in MBSE applications by setting, we would like to note that many rural communities lack hospitals, and the rural healthcare system comprises a variety of components, such as free-standing emergency departments, community health workers, or even increased provision of healthcare services at local health departments. Therefore, future applications of MBSE tools in rural settings should include other types of healthcare providers beyond the hospital.

The implications of the lack of direct references to MBSE, and more generally to the use of systems engineering in rural healthcare disaster preparedness, is twofold. First, relatively little research has been done in general on rural disaster preparedness (Manley et al. 2006; Cliff 2007; Swain 2012). Second, even less research has been published using a formal systems engineering approach for planning or preparing for disasters, particularly using computer simulation. While several publications considered the conceptual use of systems engineering and MBSE, they were eliminated from consideration, as they did not present a model or direct application.

### Limitations

There are some limitations to this scoping review to note. First, because we did not conduct a quality assessment of the studies, we are unable to discuss the quality of the simulation studies in this field. This action may have resulted in the inclusion of poor-quality studies that compromise the quality of this scoping review. Second, while we included a variety of databases to capture gray literature in this field, we did not include all such sources (for example, Google Scholar) due to the overwhelming volume of search results from these types of databases. Furthermore, our screening process was limited to English-language publications, which may have resulted in the exclusion of studies relevant to our research questions that were published in other languages. Finally, as with all systematic reviews, publication bias and selective reporting in the published literature and within the included studies are possibilities, which includes selective reporting of studies with positive outcomes (that is, successful simulations) or incomplete reporting of simulation details.

Despite these limitations, this scoping review has several important strengths. We used several techniques to ensure that all relevant literature was identified and that the results of this review would reflect the current state of the application of MBSE methods to disaster contexts in rural healthcare settings. We found no other systematic or scoping reviews on this topic in the literature. We used a standard framework to ensure that the results were methodologically sound, precise, and transparent. Our comprehensive approach included consultation with subject matter librarians, search term pilot testing, use of several appropriate electronic databases with few search limits, and hand-searching of included publications’ reference lists. Finally, we used two independent reviewers throughout the selection and data charting process to reduce the risk of selection bias.

## Conclusion

Model-based systems engineering methods, including computer simulations, have been shown to be important tools in preparedness and planning in other healthcare settings and disaster management applications. Model-based systems engineering can provide insights for disaster preparedness and planning and serve as a decision-support tool in resource-limited settings, such as rural areas. Understanding how these methods have been used in recent rural healthcare disaster contexts can support continued expansion of the use of these tools. We found six studies that utilized these methods in applications that focused on or included rural healthcare settings. Most of these were used to support preparedness and planning and focused on hospital settings. However, this scoping review identified a gap in the research regarding the application of an MBSE approach to support rural healthcare disaster preparedness planning efforts. During this work, we also noted that systems-thinking toolsets were not in widespread use for this purpose. The limited application of MBSE and systems thinking for rural healthcare disaster preparedness planning suggests that a systems-level approach constitutes a novel, worthwhile method for developing tools and computational simulations that could help rural communities use their scarce disaster planning resources effectively to improve the efficacy of their planning efforts. There is a clear need to expand the applications of MBSE to support rural disaster preparedness and to broaden the definition of healthcare system beyond the hospital setting to better serve rural healthcare preparedness needs.

## Footnotes

1 https://osf.io/t5crm/
